# Supplementary material for: Maternal Stress and Excessive Weight Gain in Infancy
Source: Int J Environ Res Public Health. 2022 May 9;19(9):5743. doi: 10.3390/ijerph19095743 (PMC9105332; doi:10.3390/ijerph19095743)
Supplement: Supplementary file 1 [file ijerph-19-05743-s001.zip › ijerph-1660816-supplementary.pdf]

Supplementary Table S1: Missing Data for Main Exposure, Outcome and Covariate Variables

Variable		N (%) Missing		
	3 months	6 months	9 months	12 months
Maternal				
Marital status	135 (20.3)	175 (26.3)	211 (31.7)	203 (30.5)
Unemployed	135 (20.3)	175 (26.3)	211 (31.7)	202 (30.3)
Low income	154 (23.1)	184 (27.6)	216 (32.4)	204 (30.6)
Household number	139 (20.9)	184 (27.6)	223 (33.5)	206 (30.9)
PSS score	272 (40.8)	295 (44.3)	292 (43.8)	312 (46.9)
Infant				
Rapid weight gain from baseline	N/A	231 (34.7)	263 (39.5)	256 (38.4)
Weight for length z-score	153 (23.0)	198 (29.73)	232 (34.8)	217 (32.6)

Supplementary Table S2: Associations Between Variables and Missingness for the Identification of Auxiliary Variables to be Used for Multiple Imputation

Variable	Not Flagged for Missing	Flagged for Missing	P Value
Maternal			
Age	28.1±5.8	26.2±5.4	<0.0001*
Race (Black)	64.9%	79.85%	<0.0001*
Ethnicity (Hispanic)	7.5%	5.2%	0.0137*
Overweight/obese	63.4%	59.1%	0.0280*
Prenatal diet quality (AHEI)	42.1+/-10.2	42.7 +/-11.6	0.5390
Married/living with partner	62.0%	51.0%	<0.0001*
Highschool or higher	55.3%	48.1%	0.0002*
Unemployed	46.0%	42.5%	0.1955
Low income (<$20,000/year)	52.9%	56.3%	0.2108
Household number	3.6±2.1	3.4±1.6	0.2196
Infant			
Gestational age at birth	38.7±1.5	38.4±1.7	0.0620
Weight for length z-score	0.4±1.1	0.5±1	0.2070
Birthweight for gestational age z-score	-0.3±1	-0.3±0.9	0.6834
Race (Black)	61.8%	77.3%	<0.0001*
Ethnicity (Hispanic)	11.5%	6.7%	<0.0001*
Infant sex (male)	50.2%	52.5%	0.2281
Total weeks breastfed	17.9±19.8	10.5±14.8	<0.0001*

Independent t-tests were used to compare normally distributed continuous variables, Wilcoxon rank sum test for non-normally distributed continuous variables, and Chi Square tests for categorical variables.

\

Supplemental Table S3: Baseline Demographic Characteristics of the Nurture Cohort of Infants with and Without Rapid Weight Gain* from 3 to 12 months and Unadjusted Risk Ratios.

Variables<br>N (%) or mean ±SE	Not Rapid<br>(n=227)	Rapid<br>(n=214)	RR<br>Exp(β)	95% CI
Maternal				
Age	27.5±0.3	27.0±0.4	1.0	1.0-1.0
Race (Black)	256(72.6)	220(70.2)	0.9	0.8-1.1
Ethnicity (Hispanic/Latinx)	19(5.3)	24(7.9)	1.2	0.9-1.7
Overweight or Obese Pre-pregnancy	199(56.4)	194(62.0)	1.1	0.9-1.4
Prenatal diet quality (AHEI)	42.0±0.7	42.5±0.7	1.0	1.0-1.0
Married or living with partner	203(57.6)	177(56.4)	1.0	0.8-1.2
Highschool degree or greater	277(78.6)	254(80.9)	1.1	0.8-1.4
Unemployed (looking for work)	68(19.4)	57(18.3)	1.0	0.7-1.2
Low Income(<20,000/year)	215(61)	184(58.6)	0.9	0.8-1.2
Number of people living in the home	3.6±0.1	3.5±0.1	1.0	0.9-1.0
Infant				
Gestational age at birth	38.4±0.1	38.8±0.1	1.1	1.0-1.1
Birth weight for GA z-score	-0.5±0.1	-0.1±0.1	1.2	1.1-1.3
Small for GA (<10th%ile)	43(12.3)	22(7.0)	0.7	0.5-1.0
Large for GA (>90th%ile)	29(8.3)	36(11.5)	1.2	0.9-1.6
Sex Male	189(53.7)	152(48.4)	0.9	0.7-1.1
Weeks Breastfed	16.2±1.1	13.0±1.0	1.0	1.0-1.0
Breastfed 6 months or greater	86(24.5)	64(20.3)	0.9	0.5-1.0
Breastfed 12 months	58(16.4)	32(10.3)	0.7	0.5-1.0

Values were calculated with log-binomial models

*Rapid weight gain defined as a change in WFL z-score >0.67 representing upward crossing of percentile bands on WHO growth chart

Perceived Stress Scale
The questions in this scale ask you about your feelings and thoughts during the last month. In each case, you will be asked to indicate by circling how often you felt or thought a certain way.

0 = Never 1 = Almost Never 2 = Sometimes 3 = Fairly Often 4 = Very Often

1. In the last month, how often have you been upset because of something that happened unexpectedly?
2. In the last month, how often have you felt that you were unable to control the important things in your life?
3. In the last month, how often have you felt nervous and “stressed”?
4. In the last month, how often have you felt confident about your ability to handle your personal problems?
5. . In the last month, how often have you felt that things were going your way?
6. the last month, how often have you found that you could not cope with all the things that you had to do?
7. In the last month, how often have you been able to control irritations in your life?
8. In the last month, how often have you felt that you were on top of things?
9. In the last month, how often have you been angered because of things that were outside of your control?
10. In the last month, how often have you felt difficulties were piling up so high that you could not overcome them?
